# Impact of N-decyl-nicotineamide bromide on copper corrosion inhibition in acidic sulfate containing environment: Electrochemical and piezoelectrochemical insights

**DOI:** 10.1016/j.heliyon.2024.e40184

**Published:** 2024-11-07

**Authors:** Gyöngyi Vastag, Ilona Felhősi, Milan Vraneš, Abdul Shaban

**Affiliations:** a University of Novi Sad, Faculty of Sciences, Department of Chemistry, Biochemistry and Environmental Protection, Trg Dositeja Obradovića 3, 21000, Novi Sad, Serbia; b Research Centre for Natural Sciences, Magyar tudósok körútja 2, 1117, Budapest, Hungary

**Keywords:** Copper corrosion, Inhibition, Ionic liquids, N-decyl nicotinamide bromide, QCM-I

## Abstract

This work investigates the inhibition effect and adsorption properties of a new tailor-made synthesized model molecule of ionic liquids, namely N-decyl nicotinamide bromide [C_10_Nic]Br in an acidic 0.1 M Na_2_SO_4_ solution (pH = 2.7) against the corrosion of copper. Electrochemical methods (*ac* electrochemical impedance spectroscopy and *dc* potentiodynamic polarization), and piezoelectric method (quartz crystal microbalance with impedance analysis (EQCM-I) were applied to study the corrosion protection performance of the inhibitor. Electrochemical measurements have indicated favorable corrosion inhibition performance of [C_10_Nic]Br. The corrosion inhibition efficiency increases with the increase of inhibitor concentration, at a [C_10_Nic]Br concentration of 10^−3^ M the efficiency reaches 93 %. The inhibitor adsorption slightly differed from the ideal Langmuir adsorption isotherm. [C_10_Nic]Br can be considered to be a mixed-type inhibitor. The inhibition efficiency was found to be time-dependent. In the presence of the highest 10^−3^ M inhibitor concentration the formation of the maximum protective effect of the inhibitor layer takes several hours, the maximum value of polarization resistance was 8.5 kΩ cm^2^ after 5 h. The copper dissolution and the inhibitor adsorption were also monitored by real-time changes in mass and viscoelasticity determined by QCM-I. It was obtained that the inhibitor adsorption on the copper surface leads to a decrease in copper dissolution and an increase in viscoelasticity. The layer on the copper surface becomes softer due to the complex between the inhibitor and the corrosion products on the surface.

## Highlights

• [C_10_Nic]Br acts as a mixed-type inhibitor against Cu corrosion under acidic conditions.
• Corrosion inhibition mechanism of [C_10_Nic]Br is achieved by its adsorption via anions on the Cu surface.
• Cu dissolution was hindered, and the viscoelasticity of the Cu surface was changed due to inhibitor adsorption.
• Good inhibition is recorded in the initial time but later on the characteristics of the surface oxide layer appear slowly.

## Graphical abstract

**Figure:**
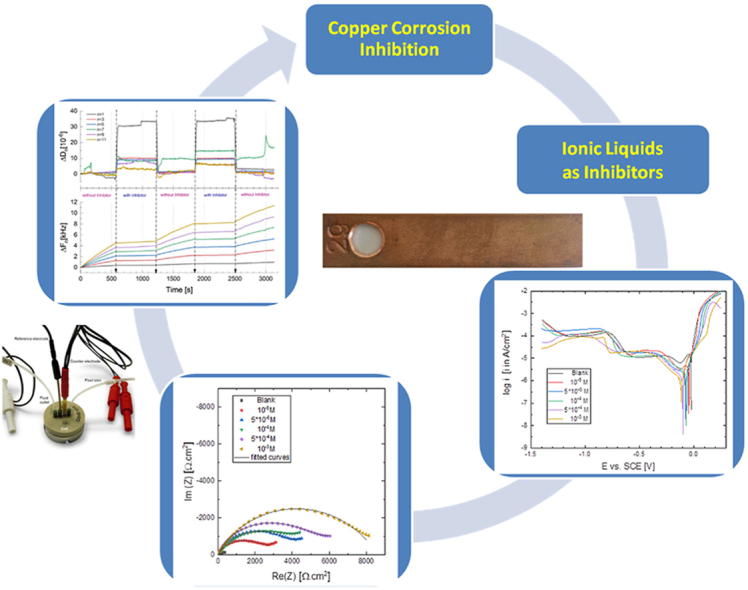

## 1 Introduction

Corrosion inhibitors are widely used in various corrosive media to protect metals against corrosion. In general, the main disadvantages of using “classical” corrosion inhibitors are their toxicity, non-biodegradability, inadequate solubility and persistence under normal working conditions. Modern research therefore strives to find metal corrosion inhibitors that, in addition to the basic properties expected of an inhibitor (high efficiency, temperature- and time stability), also meet the increasingly strict requirements of sustainable development (low-toxicity, biodegradability). In numerous investigations, different natural products and medicaments were investigated as potential green and low-cost corrosion inhibitors [[1], [2], [3], [4]]. In many cases, such environmentally friendly compounds are unstable in alkaline and acid solutions, as well as during high-temperature applications [5]. On the other hand, the use of natural products as corrosion inhibitors requires their purification, which in some cases involves the use of large amounts of organic solvents, increasing costs and limiting the possibility of their use due to increased environmental pollution [6].

Another approach to the development of new types of corrosion inhibitors is the tailor-made synthesis of molecules. This group includes ionic liquids, where it is possible to influence the physical properties (hydrophobicity, viscosity, density), chemical characteristics (solubility, toxicity) and biological behavior of ionic liquids [7] by combining different types of anions and cations (mainly of large organic origin). Ionic liquids, as low melting point (lower than 100ᴼ C) compounds, are widely used in various industries due to their several excellent properties, such as lower volatility, non-toxic nature, chemical and thermal stability, good solubility in polar solvents, less hazardous effect on the environment etc. [5,6,[8], [9], [10]]. Recognizing its tailor-made and mostly “green” properties, the scientific community is increasingly interested in the possibility of using ionic liquids as a potential inhibitor of metal corrosion in environments with different aggressiveness, as an alternative to toxic organic and inorganic compounds [8,[11], [12], [13], [14], [15], [16]].

Zunita and coworkers [17] have made a classification of the types ILs as corrosion inhibitors. Either based on interaction types they divide them into neutral, acidic, and basic ILs, or based on the properties of cations and anions it distinguishes them amphoteric, functionalized, protic, poly, and bio ILs. All the mentioned types of ionic liquids have been tested as potential corrosion inhibitors of various metals in corrosive environments of different aggressiveness [17]. The achieved inhibitory efficiency of ILs varied as expected, depending on the type of applied ILs, the used concentration, as well as on the metal and corrosion environmental properties. In most cases, ILs behaved as mixed corrosion inhibitors, where their protective properties manifested through adsorption on the metal surface obeying a pattern related to the Langmuir isotherm [8,17,18].

Copper (Cu) has good corrosion characteristics in weakly acidic, neutral, and weakly basic environments thanks to the passive layer. In an acidic environment, due to the increased solubility of Cu_2_O, Cu corrosion intensifies, and it is necessary to protect it against corrosion. Copper is often protected from corrosion in acidic media with corrosion inhibitors; mostly organic compounds with nitrogen- or sulfur-containing functional groups are used, of which azoles are the most common. Ongoing research focuses on identifying new ILs with improved corrosion inhibition properties for Cu [8,[11], [12], [13],^19^]. Ionic liquids can be applied as corrosion inhibitors for Cu as coating agents, corrosion inhibitor formulations [11,20], and green corrosion inhibitors, [6,9,16]. The most commonly used ionic liquids as inhibitors against copper corrosion are the imidazolium based ionic liquids [8,11,12].

The aim of this work was to investigate N-decyl nicotinamide bromide ([C_10_Nic]Br), as a model molecule of ionic liquids containing long hydrophobic alkyl chain and hydrophilic N-containing functional group as a potential new inhibitor against copper corrosion in acidic 0.1 M Na_2_SO_4_ solution (pH = 2.7). The synthesis of the molecule series of N-alkyl nicotinamide-based compounds and the influence of side-chain length on antifungal efficacy were studied previously [21]. Preliminary tests were carried out with N-butyl-nicotinamide as a corrosion inhibitor, where a 73 % inhibition efficiency against copper corrosion was achieved in an acidic solution (pH = 3) [13]. In this article, we characterized the corrosion inhibition efficiency and inhibition mechanism of [C10Nic]Br, a member with a longer alkyl chain of N-alkyl-nicotinamides, by using electrochemical methods (AC electrochemical impedance spectroscopy (EIS) and DC potentiodynamic polarization). The concentration- and time-dependence of inhibition was also determined.

A piezoelectric method, quartz crystal microbalance with impedance analysis (QCM-I) was also used to study copper corrosion and its inhibition by [C_10_Nic]Br, which method provides valuable data about mass changes, viscoelastic properties, and the overall dynamics of corrosion processes. The real-time monitoring of mass changes provided information on the corrosion rate enabled the identification of different corrosion stages, and the characterization of corrosion products formed on the copper surface. The changes in mass and viscoelastic properties accuring due to addition of the inhibitor to the corrosive environment were also investigated and interpreted.

## 2 Materials and methodology

### 2.1 Inhibitor molecule formula and figure

**Scheme 1:**
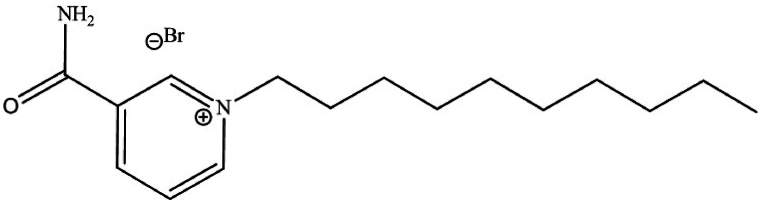
Chemical structure of the investigated ionic liquid [C_10_Nic]Br.

Scheme 1 shows the structure of the used N-decyl nicotinamide bromide ionic liquid ([C10Nic]Br). The synthesis and analytical characterization of the newly synthesized inhibitor compound was published previously elsewhere [21].

### 2.2 Applied chemicals

The corrosive solution was prepared by dilution of analytical grade Na_2_SO_4_ by Milli-Q deionized water. The pH of the used 0.1M Na_2_SO_4_ solution was adjusted to 2.70 using diluted H_2_SO_4_ acid before carrying out the experiments. The concentration of the tested [C_10_Nic]Br alternated from 1 × 10^−5^, 5 × 10^−5^, 1 × 10^−4^, 5 × 10^−4^, and 1 × 10^−3^ M in the electrochemical measurements. For the QCM-I tests the concentration of 1 × 10^−3^ M was used.

### 2.3 Experimental methods

#### 2.3.1 Electrochemical measurements

A three-electrode cell was used for the electrochemical experiments and placed in a Faraday cage. The counter electrode was a platinum mesh, the reference electrode was a saturated calomel electrode (SCE), and the working electrode was a DHP (deoxidized high phosphorus) Cu rod (99.9 %) with a cross-section of 0.785 cm^2^, embedded in epoxy resin. Before measurements the working electrode was mechanically polished with successive grades of silicon carbide polishing paper from 220 down to grade 4000.

All electrochemical measurements (potentiodynamic polarization, cyclic voltammetry and EIS) were performed using a Solartron 1286 Potentiostat and a Solartron 1250 Frequency Response Analyser (FRA). Instrument control and fitting of the measured data was performed by using CorrWare/Corrview and ZPlot/Zview softwares. During the first 30 min of immersion the open circuit potential was recorded. Potentiodynamic polarization measurements were started after 30 min of immersion in all cases to ensure the steady-state open circuit potential. The polarization curves of anodic and cathodic scans were recorded by two independent measurements, starting the scans from the corrosion potential. The scan rate was 10 mV/min (0.166 mV/s). Cyclic voltammetry measurements were taken by scanning the potential from −1400 mV to 100 mV at 20 mV/s scan rate. Electrochemical Impedance Spectroscopy was used to monitor the time dependence of corrosion during 20-h immersion, using a series of measurements in which OCP and EIS measurements were repeated alternatively. Impedance spectra were recorded at OCP using a 10 mV effective amplitude sinusoidal signal with 10 points per decade over frequencies ranging from 10,000 to 0.01 Hz. The OCP was monitored for 30 min, (changes in the OCP should be less than 5 mV/5 min), before starting individual impedance spectra. In all electrochemical measurements, the applied potential was referred to the SCE reference electrode, unless otherwise mentioned.

#### 2.3.2 QCM-I measurements

**Fig. 1:**
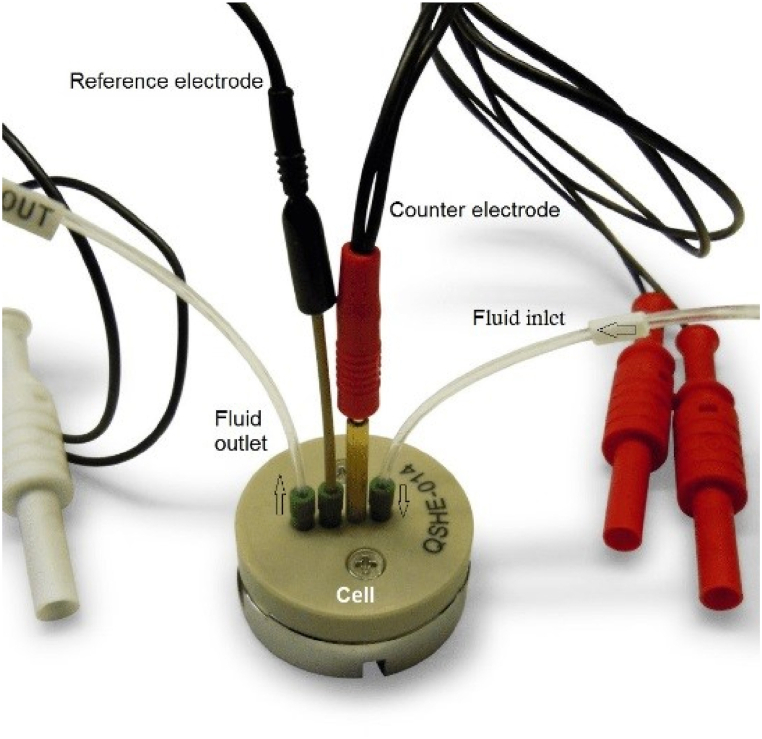
Fluidic electrochemical cell applied in the EQCM-I measurements.

The applied QCM-I setup was a QCM008M01dll instrument from MicroVacuum Ltd. (Budapest, Hungary). The instrument resonance, dissipation, and mass sensitivities in liquid are 2 × 10^−1^ Hz, 0.1 × 10^−6^, and ≤1 ng/cm^2^, respectively. The volume of the attached electrochemical flow-cell was ∼40 μl. The QCM resonator used in this investigation was an AT-cut crystal with a 14 mm diameter and 5 MHz fundamental resonance frequency. The fabricated gold layer was deposited on a TiO_2_ layer. The *in-situ* experiments were performed at a flow rate of ≈1 μl/s maintained by a peristaltic pump. The resonance frequency and dissipation shifts were recorded with the time resolution of 6.2 s for each selected over-tone (the overtone numbers were n = 1, 3, 5, 7, 9 for the frequencies of 5, 15, 25, 35, 45 MHz, respectively), using the BioSence 3.25.18 software. The used QCM-I cell setup, illustrated in Fig. 1, had a platinum disk as a counter electrode silver/silver chloride as a reference electrode, and the resonator gold surface as the working electrode [22].

Before the electrochemical measurements, the gold resonator electrode was cleaned with isopropanol and rinsed with ultrapure grade Milli-Q water (resistivity of 18MΩ·cm). A three-electrode cell was used for the Cu deposition, with the QC gold as the working electrode, silver/silver chloride (Ag/AgCl) as the reference electrode, and the platinum disk as the counter electrode, at a current density of 1000 μA/cm^2^ for 170 s, using a deposition bath consisting of 0.5 M CuSO_4_, 1 M C_2_H_5_OH, and 0.5 M H_2_SO_4_, as previously described [23].

## 3 Results and discussion

### 3.1 Surface coating characterization

**Fig. 2:**
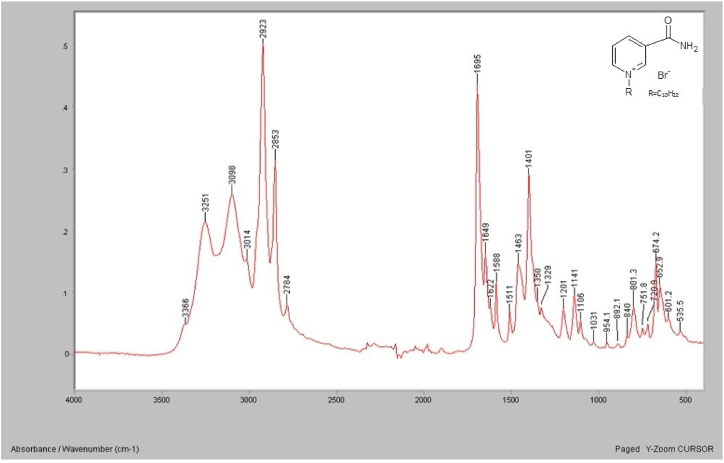
FTIR spectrum for the N-decyl nicotinamide bromide.

A sample of the [C_10_Nic]Br was analyzed by FTIR and the spectrum is shown in Fig. 2. The spectrum shows three main functional groups of C-H, C-G alkene, and C=C. By examining the spectrum: The C-H stretching-band hydrocarbons in a range of 2923 cm^−1^, where it can be seen in the C-H alkene in 3215-3098 cm^−1^ range and C=C bond at 1665 cm^−1^ and are of either weak or medium intensity. The band greater than 3000 cm^−1^ for the = C–H stretch and the several bands lower than 3000 cm^−1^ for –C–H stretch (alkanes). The C=C stretch band is at 1665 cm^−1^. Bands for C–H scissoring (1465 cm^−1^) and methyl rock (1378 cm^−1^) are marked. These bands are not definite to an alkene and are usually not distinguished since they exist in nearly all organic molecules (in the fingerprint region). The bands at 801 and 674 cm^−1^ are related to the bending of the C-H.

### 3.2 Electrochemical investigation of the inhibitor effect

#### 3.2.1 OCP measurement in time

**Fig. 3:**
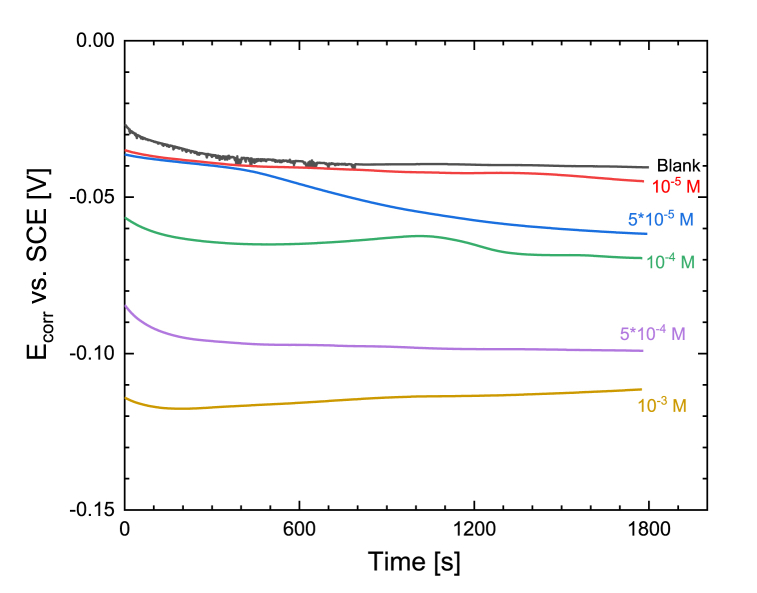
Change of corrosion potential in the first 30 min of immersion as a function of [C_10_Nic]Br inhibitor concentration.

The change of corrosion potential of Cu in time for 30 min immersed in aerated 0.1M Na_2_SO_4_ solution (pH was adjusted to 2.7) without and with the addition of N-decyl nicotinamide bromide ([C_10_Nic]Br) is presented in Fig. 3. After a slight change in the first minutes, the corrosion potential reaches its steady-state at about 5–20 min after immersion. The slight change of potential at the beginning of immersion may be attributed to the formation of an adsorbed film and thin Cu(I)oxide on the freshly polished copper. The presence of [C_10_Nic]Br inhibitor influences the corrosion potential (*E*_*corr*_), which shifts toward the cathodic direction. There is a correlation between the concentration of [C_10_Nic]Br inhibitor, and the *E*_*corr*_ values, the larger the concentration, the larger the shift of *E*_corr_.

#### 3.2.2 Potentiodynamic polarization behavior

**Fig. 4:**
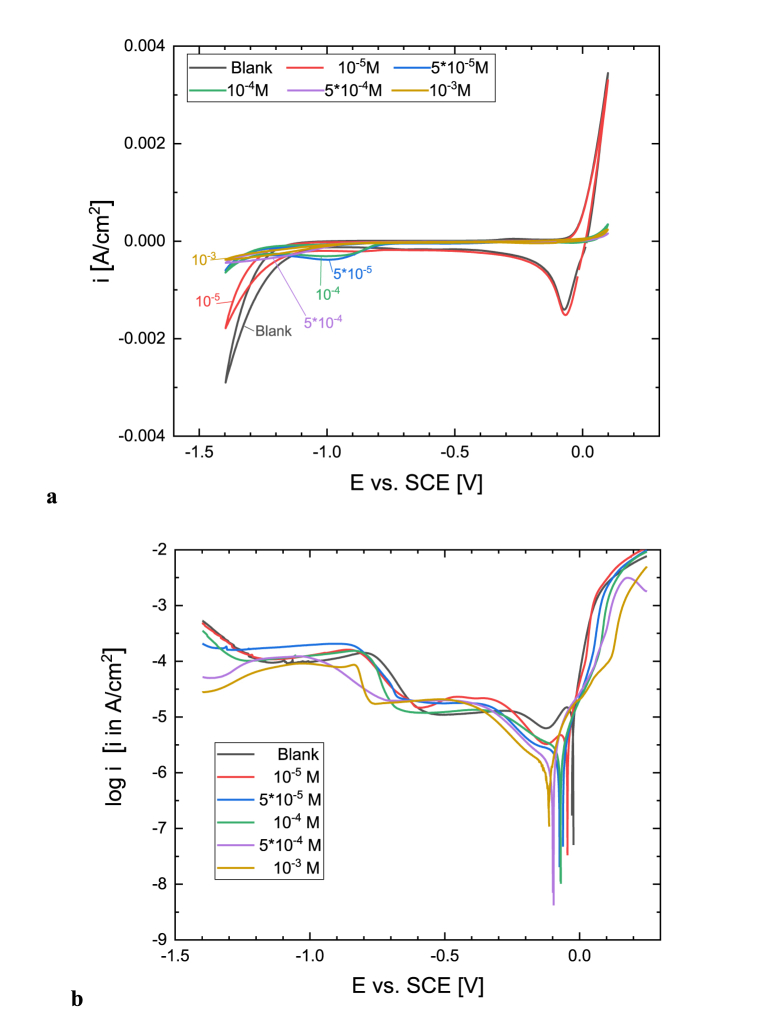
a) Cyclic voltammetry curves (scan rate 20 mV/s, depicting the 5th cycles) and **b**) Polarization curves (scan rate 10 mV/min) for Cu in acidic 0.1 M Na_2_SO_4_ solution (pH = 2.7) containing different concentrations of [C_10_Nic]Br inhibitor.

Cyclic voltammograms were recorded to characterize the corrosion of copper in an acidic sulfate solution in the presence of an inhibitor. Voltammograms are shown in Fig. 4a and the results indicate that the presence of inhibitor has an effect of reducing the anodic current density that is particularly pronounced at higher concentrations. This phenomenon can be explained by the formation of a protective adsorption layer of inhibitor. In the absence and with 10^−5^ M [C_10_Nic]Br inhibitor concentration a cathodic current peak is observed at the polarization curve at potential −70 mV vs. SCE, which is attributed to the reduction of cupric species formed by the corrosion process as presented by several authors [[24], [25], [26], [27]]. In the presence of [C_10_Nic]Br inhibitor with increasing concentration, this current peak decreases, and at 5 × 10^−4^ and 10^−3^ M concentrations, disappears, proving a good protection effect against copper corrosion. Further increasing the cathodic polarization, the diffusion-limited oxygen reduction domain characterizes the voltammograms. Another cathodic current peak is also visible at around −1 V vs. SCE potential which is related to the sulfate desorption process [26,27]. At potentials below −1.2V, the current increase corresponds to the hydrogen evolution, which is an activation-controlled reaction in an uninhibited solution. The hydrogen evolution process is also hindered by the adsorption of the inhibitor.

**Fig. 5:**
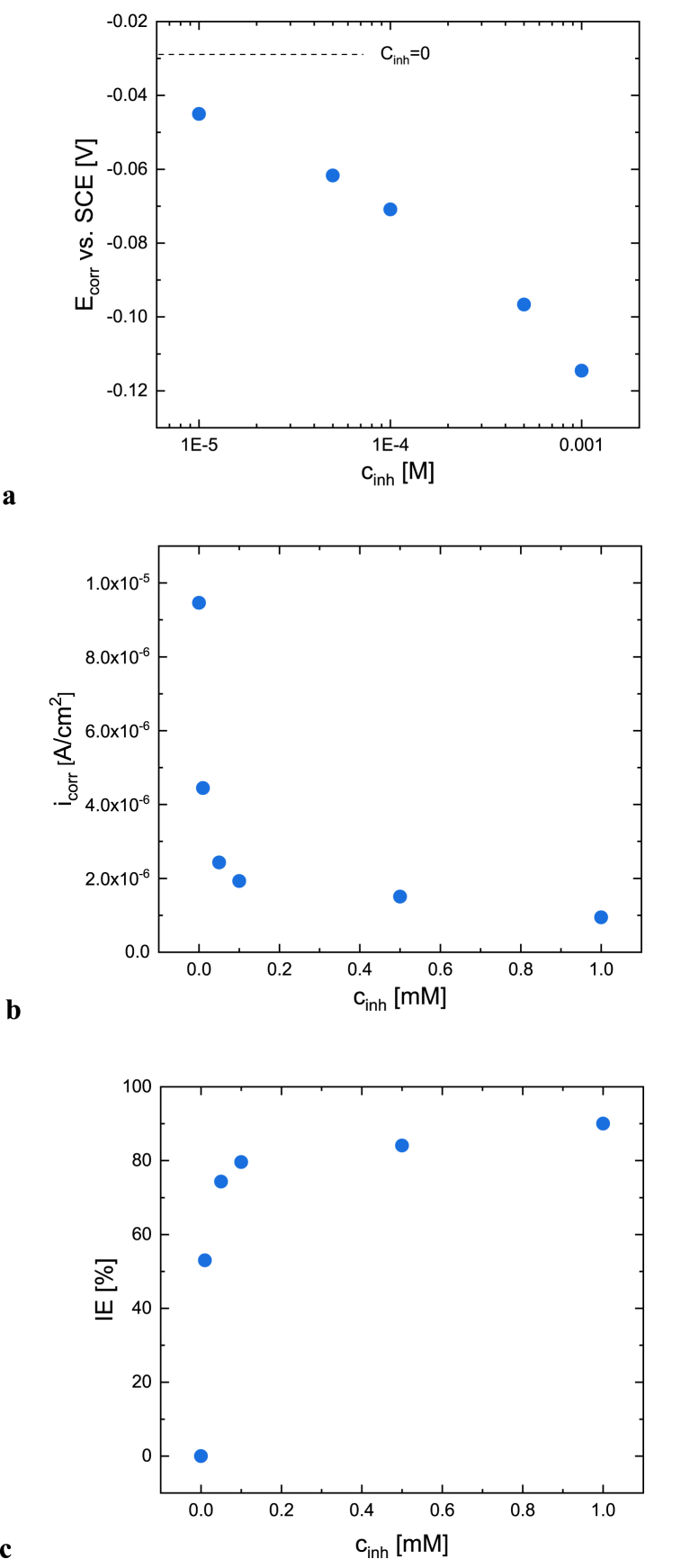
The (a) corrosion potential, (b) corrosion current density, and (c) inhibitor efficiency of copper in acidic 0.1 M Na_2_SO_4_ solution (pH = 2.7) as a function of [C_10_Nic]Br inhibitor concentration.

Potentiodynamic polarization measurements were started after 30 min of immersion to ensure the steady-state potential. Fig. 4b shows the anodic and cathodic polarization curves measured in aerated acidic 0.1M Na_2_SO_4_ solution (pH = 2.7) in the absence and presence of various concentrations of [C_10_Nic]Br. The *E*_*corr*_ shifts towards more negative values with increasing inhibitor concentration (Fig. 5a). *i*_*corr*_ values, determined only from the anodic part of polarization curves, also decrease with increasing concentration (Fig. 5b). The anodic range of the polarization curve shows that active copper dissolution is taking place in uninhibited acidic sulfate solution, the Tafel slope is 37 mV. The presence of [C_10_Nic]Br inhibitor remarkably decreases the anodic current densities. In the absence and with 10^−5^ M [C_10_Nic]Br inhibitor concentration a small cathodic current peak is observed at the polarization curve at potentials 20–30 mV more negative than *E*_*corr*_, in agreement with voltammograms, which is attributed to the reduction of Cu(I) formed by the corrosion process. In an uninhibited solution, the charge determined from this current peak is around 2.4 mC/cm^2^, corresponding to 2.5 × 10^−8^ mol/cm^2^ surface excess concentration. At 5 × 10^−4^ M and 10^−3^ M concentrations this cathodic current peak disappears. Consequently the [C_10_Nic]Br inhibitor can be considered to be a mixed-type inhibitor, inhibiting the rate of both anodic and cathodic processes.

The corrosion potential, the corrosion current density, and inhibitor efficiency values determined from polarization curves are tabulated in Table 1. The cathodic region of the polarization curve was not taken into account for determining the *i*_*corr*_, since the small cathodic polarization region is described by the superposition of two processes, oxygen reduction, and Cu(I) ion reduction. In the anodic range of the polarization curve, the inhibitor-free solution shows activation-controlled copper dissolution. For inhibitor-containing solutions, deviation from ideal activation control is visible, but the calculation of the *i*_*corr*_ values was also possible. *i*_*corr*_ decreases with increasing inhibitor concentration as seen in Fig. 5b. The inhibitor efficiency (η) was also determined from the *i*_*corr*_ values, according to the following equation:

(1) $$\eta \left(\%\right)=\frac{i_{0}-i_{inh}}{i_{0}}∙100\%\text{,}$$

where *i*_*o*_ is the *i*_*corr*_ of Cu in an inhibitor-free solution, and *i*_*inh*_ is the *i*_*corr*_ of Cu in the presence of an inhibitor. The results presented in Fig. 5c reveal the good inhibitory effect of [C_10_Nic]Br in the short-time test (30 min). The inhibition efficiency is concentration-dependent, reaching 90 % at [C_10_Nic]Br concentration of 10^−3^ M.

**Table 1:** Summary of the E_corr_, b_a_, i_corr,_ and η% values determined from the potentiodynamic polarization curves of copper in acidic 0.1 M Na_2_SO_4_ solution (pH = 2.7) in the presence of [C_10_Nic]Br inhibitor with different concentrations.

**C**_**inh**_**(M)**	**E**_**corr**_ (**mV)**	**b**_**a**_**(mV)**	**i**_**corr**_**(A/cm**^**2**^**)**	**η (%)**
0	−28.3	37.0	7.43 × 10^−6^	–
10^–5^	−45.0	46.8	3.5 × 10^−6^	53.0
5 × 10^−5^	−61.7	62.4	1.9 × 10^−6^	74.3
10^–4^	−70.9	59.5	1.5 × 10^−6^	79.6
5 × 10^−4^	−96.6	55.5	1.2 × 10^−6^	84.1
10^–3^	−114.5	55.1	7.4 × 10^−7^	90.0

If we assume that the inhibitor efficiency is equal to the surface coverage, we can determine the slope *m* of the following general equation of the Langmuir isotherm model:

(2) $$\frac{C}{\theta }=\frac{1}{K_{ads}}+mc\text{,}$$

*m* will be equal to 1.107, which deviates from the value of unity. Therefore, we can state that the adsorption of inhibitor deviates from the ideal Langmuir isotherm [28]. The reason for this is most probably that the *i*_*corr*_ values are not only influenced by inhibitor adsorption but also by the amount of Cu(I)-oxide formed on the surface. In other words, since corrosion products are also present on the surface, the surface coverage of the inhibitor cannot be directly determined by inhibitor efficiency. The lower the inhibitor concentration, the more significant the difference resulting from oxide formation.

#### 3.2.3 Time dependence of corrosion followed by EIS

Polarization curves provide quantitative information on the corrosion process at a given time (30 min). Both the inhibitor adsorption and the corrosion are time-dependent processes. The EIS measurement uses a small (10 mV) bias that does not affect the change of corrosion over time; therefore it is suitable for monitoring the time dependence of the corrosion process. To monitor the corrosion and its inhibition, a series of measurements were applied in which the OCP and EIS measurements were repeated cyclically.

**Fig. 6:**
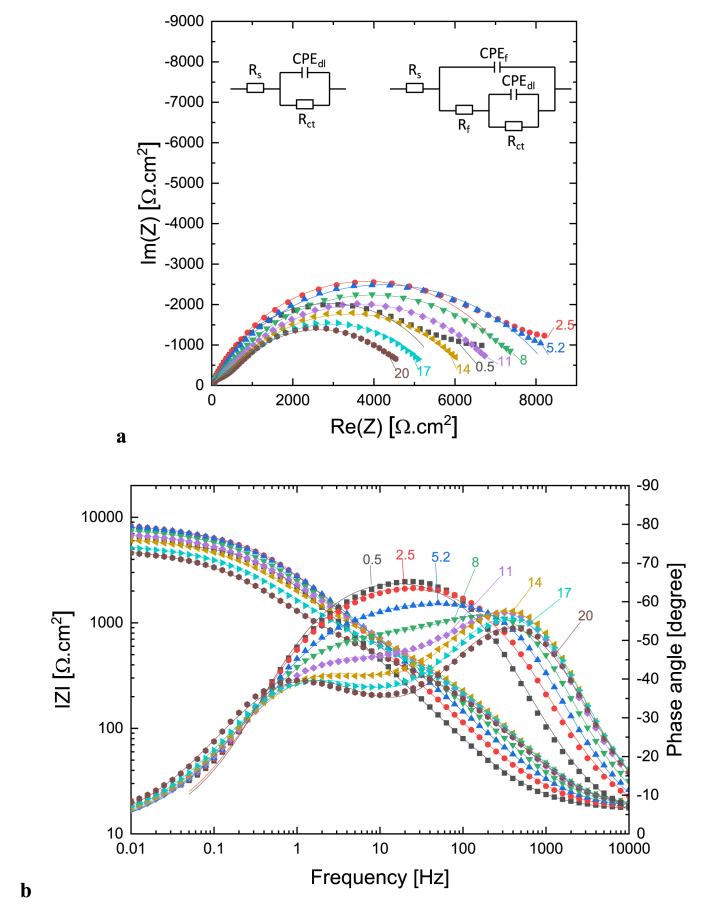
A representative of the Cu electrode impedance spectrum in 10^−3^ M [C_10_Nic]Br + 0.1M Na_2_SO_4_ solution (pH = 2.7) after different immersion times: a) Nyquist plot and b) Bode plot. Numbers indicate immersion time, symbols are measured data, and solid lines are fitted curves.

Fig. 6 shows the Nyquist (a) and Bode (b) plots of impedance spectra of Cu measured in the presence of 10^−3^ M [C_10_Nic]Br inhibitor after different immersion times. The equivalent circuits used to fit experimental impedance spectra are shown in the inset of Fig. 6a, *R*_*s*_ represents the solution resistance, *R*_*ct*_ charge transfer resistance of Cu dissolution, and *CPE*_*dl*_ is the non-ideal double layer capacitance. *R*_*f*_ and *CPE*_*f*_ values correspond to the surface film properties; electrolyte resistance inside the pores of formed oxide film, and non-ideal capacitance of the oxide film, respectively. Similar impedance characteristics were reported for inhibition of copper corrosion by several authors [[29], [30], [31], [32]]. All fitted parameters of the impedance spectra shown in Fig. 6 are provided in Supplementary Table S1, and the time dependence of fitted parameters are plotted in Fig. S1.

It can be observed in Fig. 6 that the impedance spectrum is characterized by a capacitive loop for 5 h at the beginning of the immersion. The diameter of the capacitive arc (*R*_*ct*_) exhibits an increasing trend. This indicates that the formation of the inhibitor film is continuous over a longer period. Maximum efficiency was obtained at 5 h of immersion. After this time, a second capacitive loop appears which is characteristic of the surface oxide layer, indicating the appearance and development of a porous corrosion product. The polarization resistance decreases and the *Y* value of *CPE*_*dl*_ increases with time. Consequently, after the start of corrosion, the protective effect of the inhibitor layer also begins to decrease.

Based on the time-dependence of impedance spectra, it can be said that the adsorption of the inhibitor prevents copper oxidation at the initial time, but at later times, after 5 h, the characteristics of the surface oxide layer also appear, which affects the stability of the inhibitor film, and inhibitor desorption can also be assumed. This type of time variation of the impedance spectra is typical for corrosion systems where the formation of the corrosion product is slow, taking several hours. Several authors have reported similar behavior over investigating the inhibition of copper corrosion in acidic solutions [29,30].

**Fig. 7:**
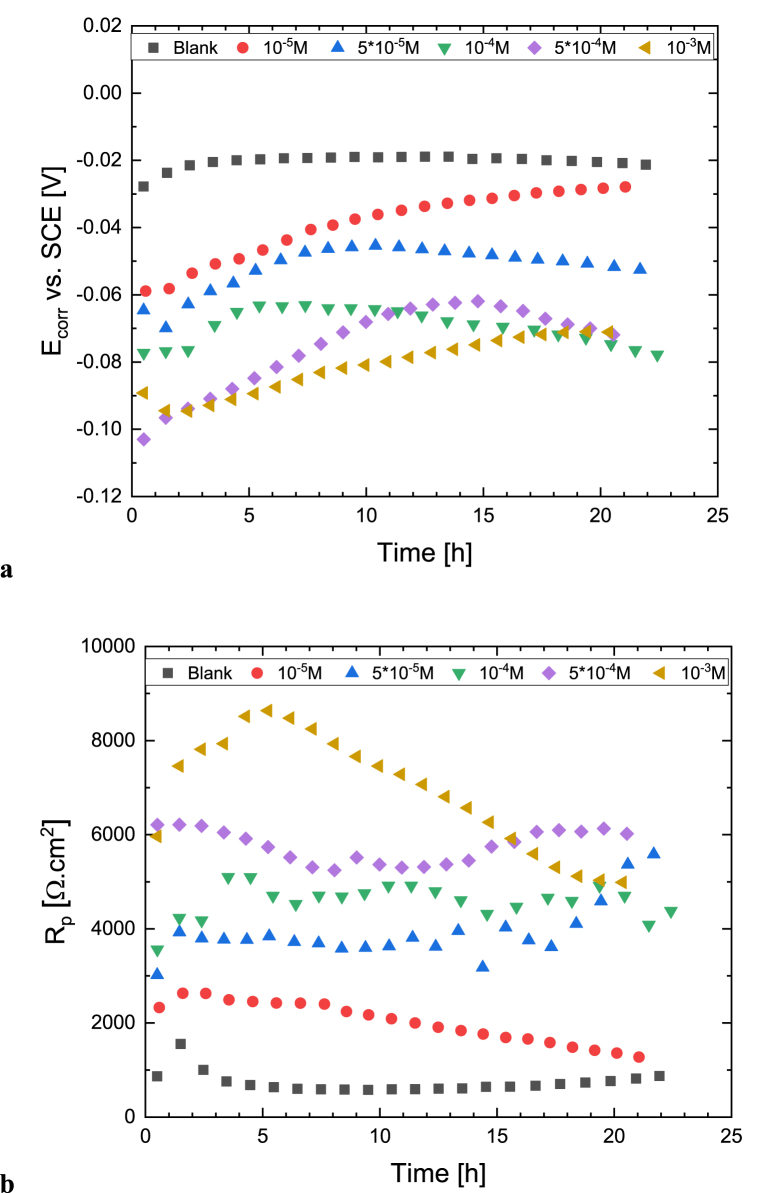
The time-dependent change of the corrosion potential (a) and polarization resistance (b) determined by the impedance spectra of Cu in acidic 0.1 M Na_2_SO_4_ solution (pH = 2.7) containing different concentrations of [C_10_Nic]Br inhibitor.

Fig. 7 shows the time-variation of corrosion potential and polarization resistance of copper measured in the presence of various inhibitor concentrations (10^−5^ to 10^−3^ M). The bias voltage in the setup was set to 0 V vs. OCP, which is measured before obtaining the impedance spectrum. The polarization resistance values (*R*_*p*_) are determined through the impedance spectra fitting and discounting the electrolyte resistance (*R*_*s*_). In agreement with the previously presented results of polarization measurements, the corrosion potential depends on the inhibitor concentration, the larger the concentration, the more cathodic the shift. The polarization resistance is largely influenced by the concentration of the inhibitor.

In an acidic inhibitor-free sulfate solution, the value of *R*_*p*_ varies between 0.6 and 1.5 kΩ cm^2^. In the presence of the highest 10^−3^ M inhibitor concentration, *R*_*p*_ is an order of magnitude higher; its value varies between 5 and 8.5 kΩ cm^2^. The value of *R*_*p*_ increases initially, its maximum value was measured after 5 h of immersion.

This can be explained by the time-dependent formation of a protective inhibitor layer that takes several hours to develop maximally. With the appearance of corrosion, the protective effect of the inhibitor layer begins to decrease.

**Fig. 8:**
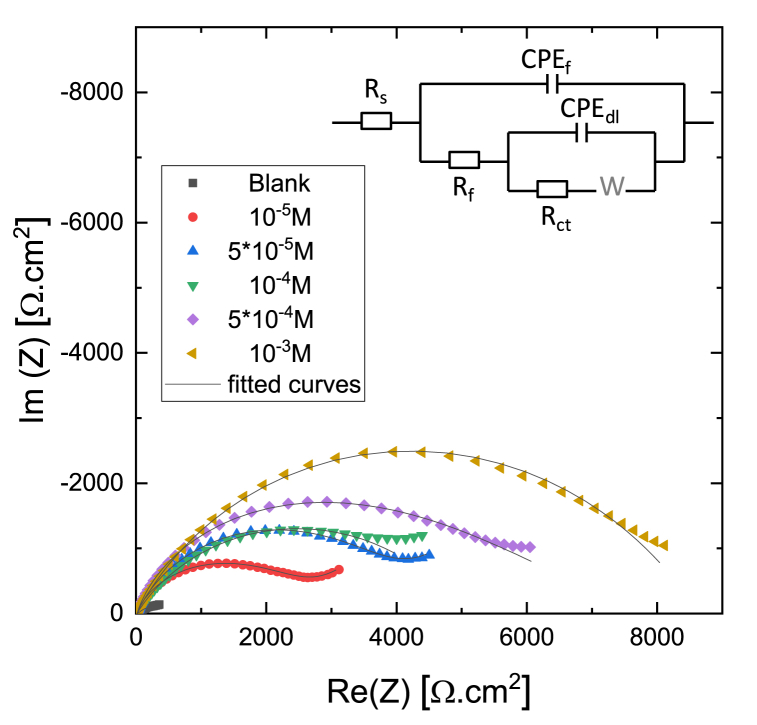
Impedance spectra of copper after 5 h of immersion time in acidic 0.1 M Na_2_SO_4_ solution (pH = 2.7) containing different concentrations of [C_10_Nic]Br inhibitor.

Fig. 8 shows the Nyquist impedance spectra of Cu measured in the presence of different inhibitor concentrations after 5 h of immersion time. The Nyquist diagrams displayed depressed semicircles which reveal that the corrosion process is under charge transfer control. Semicircles, having the center below the real axis, the so-called dispersing effect, are typical for real corroding systems. The effect of the concentration of the inhibitor can be clearly observed in the figure, as the concentration increases, the diameter of the semicircle that is the value of R_ct_ gradually increases, which indicates the improvement of the corrosion protection effect. Furthermore, similar to what was described in Fig. 6, the impedance spectra after 5 h of immersion can be characterized by two-time constants; in addition to the charge transfer process, the characteristic of the corrosion layer also appears at high frequencies. In addition, Warburg diffusion impedance can also be observed at low frequencies in the case of inhibitor-free and at lower inhibitor concentrations (<10^−4^ M), which is typical for copper dissolution in acidic solutions and indicates the Cu(I) ion diffusion from the electrode to the solution, and/or oxygen diffusion from the bulk of solution to the interface of electrode [3,4,[29], [30], [31], [32]].

The obtained EIS data from Fig. 8 are listed in Table 2. The inhibition efficiency obtained from the EIS data (*η*_*eis*_) is calculated using the charge transfer resistance data in inhibited and uninhibited solutions, respectively, using the following equation:

(3) $$\eta_{eis}\left(\%\right)=\frac{R_{p}^{0}-R_{p}^{i}}{R_{p}^{i}}∙100\text{,}$$

where *R*^o^_p_ and *R*^i^_p_ are the polarization resistance in the absence and presence of the inhibitor, respectively.

**Table 2:** Summary of EIS values determined by fitting impedance spectra recorded on a copper electrode after 5 h of immersion in acidic 0.1 M Na_2_SO_4_ solution (pH = 2.7) in the absence and presence of various concentrations of [C10Nic]Br inhibitor.

**c**_**inh**_	**R**_**s**_	**Y(CPE)**_**f**_	**n**_**f**_	**R**_**f**_	**Y(CPE)**_**dl**_	**n**_**dl**_	**W**	**R**_**ct**_	**R**_**p**_	**χ**^**2**^	**η**_**eis**_
M	Ω cm^2^	Ω^−1^ cm^−2^ s^n^		Ω cm^2^	Ω^−1^ cm^−2^ s^n^		Ω cm^2^ s^−1/2^	Ω cm^2^	Ω cm^2^		%
0	18.4	1.5∗10^−4^	0.86	25.5	4.23∗10^−3^	0.57	0.02	574	600	6.5∗10^−4^	–
10^–5^	16.0	6.4∗10^−5^	0.80	862	2.55∗10^−4^	0.47	0.0041	1865	2727	3.1∗10^−5^	78
5 × 10^−5^	27.6	4.2∗10^−6^	1	25.4	1.16∗10^−4^	0.67	0.0034	3842	3867	1.7∗10^−5^	84.5
10^–4^	19.4	2.9∗10^−6^	0.92	6.5	1.87∗10^−4^	0.64	–	5042	5048	1.1∗10^−4^	88.1
5 × 10^−4^	19.6	4.2∗10^−6^	0.85	6.2	7.19∗10^−5^	0.39	–	5737	5743	3.9∗10^−5^	89.5
10^–3^	17.0	3.2∗10^−5^	0.82	203	6.11∗10^−5^	0.58	–	8237	8440	6.1∗10^−5^	92.9

Similarly to the polarization curves shown in Fig. 4b, the concentration dependence of the corrosion inhibition effect can also be observed in the impedance spectra. As the inhibitor concentration increases, *R*_*p*_ increases, the highest value was obtained at a concentration of 10^−3^ M, producing excellent inhibitor efficiency of 93 %. The results are also consistent with the inhibitor inhibition effect value shown in Fig. 5c.

#### 3.2.4 Interpretation of QCM-I results

##### 3.2.4.1 Galvanostatic Cu deposition

**Fig. 9:**
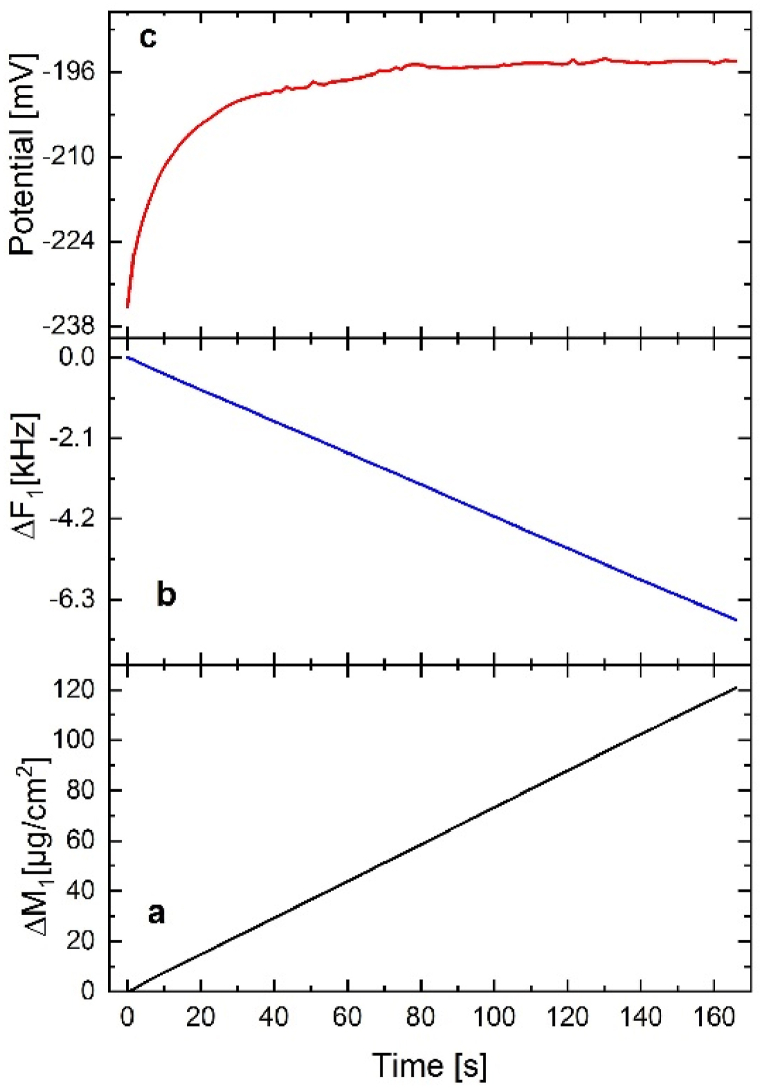
Changes of Sauerbrey mass (Δm) (a-black), changes of frequency (Δf) (b-blue), and changes of electrode potential (c-red), in time (s).

Fig. 9 shows the results of copper deposition on the resonator surface. The deposited Cu mass (Sauerbrey mass) on the resonator increased in time until it reached the final value of 120 μg (since the electrode area is estimated to be ≈ 1 cm^2^), (Fig. 9a). It is also noticed that the crystal frequency (fundamental) decreased as a result of the deposited mass, as shown in Fig. 9b. As the resonator surface was covered more and more by the deposited Cu, the electrode potential shifted closer to the Cu OCP (−196 mV vs. Ag/AgCl reference electrode), Fig. 9c).

It is worth mentioning that when estimating the Sauerbrey mass, it was assumed that the deposited surface was rigid enough [33].

##### 3.2.4.2 Change of dissipation and frequency vs. time (Δf_n_/n, ΔD_n_ and ΔFWHM_n_/n vs. t)

The extreme sensitivity of QCM-I to changes in both the mass and viscoelasticity of the load on its resonator surface in real-time and in situ enabled the examination of the dynamic corrosion and inhibition processes at the solid/solution interface. The measured parameters are the changes in oscillation frequency (Δ*f*) and energy dissipation (Δ*D*) with time at different over-tones (n = 1, 3, 5, 7, 9, 11).

When mass is adsorbed onto the resonator surface, the resonance frequency (*f*) decreases proportionally to the added mass, and vice versa. It is noted that the mass acquired includes both the mass of the materials and the water associated with the resonator surface (probably the water hydrated by the materials). Parameter (*D*) denotes the viscoelasticity of the layer on the surface. A higher *D* value designates that the layer on the surface is thick and soft, while a lower *D* refers to a more rigid and compact layer. A soft layer is usually characterized by not only the mass but also the viscosity and the elastic modulus [34,35].

Using only one overtone, which provides us with two input parameters, Δ*f*_1_ and Δ*D*_*1*_, it therefore does not provide adequate data to characterize the viscoelastic properties of the system. For different overtones (n = 1,3,5,9), the step-like curves of the normalized frequency shifts are characterized by different Δ*f*_n_ and Δ*D*_*n*_ values, therefore they provide necessary additional data for a complete understanding of the behavior of the inhibitor layer [34].

**Fig. 10:**
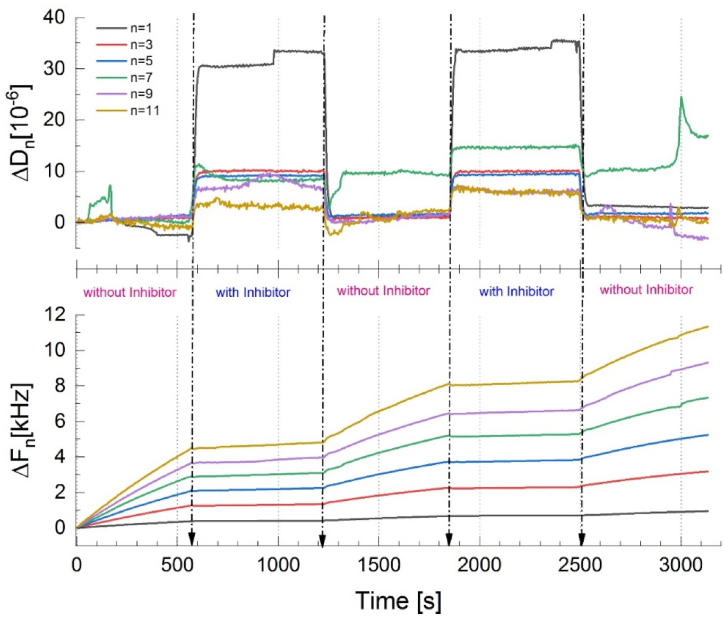
Real-time QCM-I responses at different overtones (1, 3, 5, 7, 9, 11), showing the successive changes of Δ*f*_*n*_ and Δ*D*_*n*_ with time on the injection of blank solution without and with the addition of the [C_10_Nic]Br inhibitor for 2 cycles.

Fig. 10a shows the real-time QCM-I measurements, indicating the consecutive introduction of sample solutions as specified in the figure. The injection of 0.1 M Na_2_SO_4_ solution (i.e. without inhibitor) stimulates a normalized increase in (Δ*f*_*n*_*/n)* of ≈400 Hz/600 s), and a slightly stable behavior in Δ*D* ≈ 0, for all tested overtones. The dissipation energy change (Δ*D)* for the 7th overtone was irregular compared with the other overtones thus omitted from any further treatment.

Switching to a solution containing 10^−3^ M [C_10_Nic]Br inhibitor noticeably reduced the increase rate of Δ*f*_*n*_*/n*, which corresponds to an overall decrease in the rate of mass dissolution. The dissipation energy (Δ*D)* on the sensor surface shifted to a more positive value (5-10 × 10^−6^) as a sign of the inhibitor adsorption on the resonator Cu surface.

Changing the electrolyte back to the aggressive 0.1 M Na_2_SO_4_ solution was manifested in an increase of the Δ*f*_*n*_*/n,* but not as sharp as in the first period. This suggests that the adsorbed inhibitor remains partially attached to the Cu surface thus further protecting the surface.

To quantify the effectiveness of [C_10_Nic]Br against Cu dissolution, the electrode thickness reduction rate (δ) was calculated using Faraday's law [23].

(4) δ=(Δm·3,600·24·365·10,000)/A·ρ·t

where *δ* is [mm/yr.]; *Δm* is mass loss (g), (*Δm* = − (*C).Δf*_*n*_*/n*), where *C* = sensitivity of the crystal (C = 17.7 ng cm^−2^ Hz^−1^ in our case); A is electrode surface area (cm^2^); t is time (s); *ρ* is Cu density (8.94 g cm^−3^).

The obtained electrode thickness reduction rate results, calculated using Eq. (4), and the [C_10_Nic]Br inhibition efficiencies are presented in Table 3. Please note that the applied frequency variation values were normalized as Δ*f*_*n*_*/n*.

**Table 3:** The thickness reduction rate of Cu in 0.1 M Na_2_SO_4_ without and with the addition of the [C_10_Nic]Br inhibitor.

Injected solution	Cycle 1		Cycle 2	
	Blank	+10^−3^ M [C_10_Nic]Br	Blank	+10^−3^ M [C_10_Nic]Br
Thickness reduction rate (mm/yr.)	416.25	62.44	238.12	47.32
Inhibition Efficiency (η_qcm_%)	–	85	43	88.6

Table 3 shows that the addition of the [C_10_Nic]Br inhibitor reduced the dissolution of Cu from 416.25 to 62.44 mm/year which corresponds to an inhibition efficiency of *η* = 85 % in the first cycle and 88.6 % in the second cycle. These results are in good agreement with the electrochemical results.

##### 3.2.4.3 Change of frequency vs. change of FWHM (Δf_n_/n vs. ΔFWHM_n_/n)

Quartz Crystal Microbalance with impedance analysis monitoring (QCM-I) is a powerful technique used to study thin films, including their adsorption, desorption, viscoelastic properties, and conformational changes. The analysis involves monitoring the frequency (Δ*f*) and dissipation (Δ*D*) of an oscillating quartz crystal as a function of time or other variables, such as ΔFWHM [36,37]. The relationship between Δ*f* and Δ*D* can be interpreted in the following manner: a decrease in Δ*f* (negative change) usually relates to an increase in mass loading on the resonator. If Δ*D* increases concurrently, it suggests that the adsorbed mass is viscoelastic or causes the layer to become more dissipative. In other cases, if Δ*f* decreases and Δ*D* remains relatively unchanged or decreases slightly, it suggests that the adsorbed layer is rigid and less dissipative (hard). In the same way, an increase in Δ*f* (positive change) is a sign of desorption or mass dissolution and if Δ*D* decreases, it suggests the remaining layer is more rigid or the desorbed material was viscoelastic. Significant deviations in Δ*D* without corresponding large changes in Δ*f* can indicate changes in the viscoelastic properties of the layer, such as solvent uptake (swelling) or inhibitor conformational changes without significant mass change [38].

**Fig. 11:**
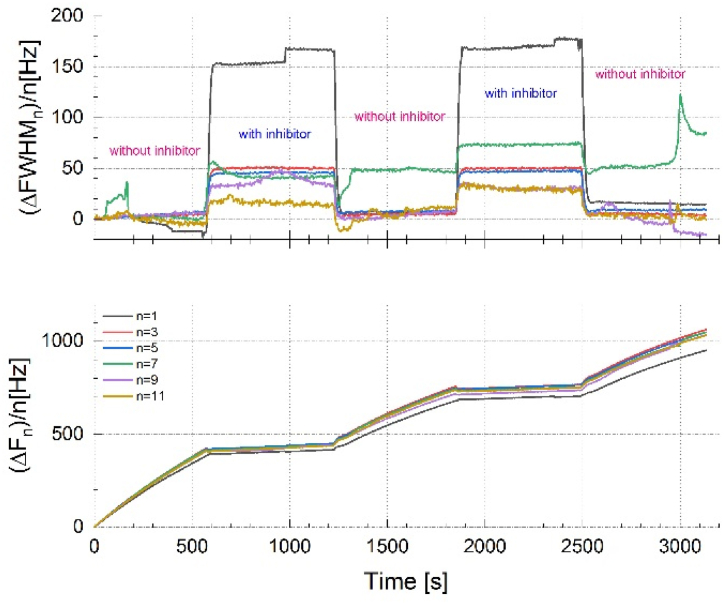
Normalized values of ΔFWHM_n_ and Δf_n_ (ΔFWHM_n_/n and Δf_n_/n) vs. time, for Cu electrode in 0.1 M Na_2_SO_4_ solution without and with the addition of 10^−3^ M of [C_10_Nic]Br inhibitor.

The ΔFWHM of the quartz-crystal sensor increases when a viscoelastic film is deposited. The results of ΔFWHM and Δ*f*_*n*_*/n* vs. time, for Cu electrode in 0.1 M Na_2_SO_4_ solution without and with the addition of 10^−3^ M of [C_10_Nic]Br inhibitor is displayed in Fig. 11. Fig. 11, as expected for a thin film, shows a great similarity to Fig. 10. The ΔFWHM behavior is similar where it shifts to a higher value to stay stable. In the meantime, the responses measured at different overtones spread out noticeably. When the blank solution was replaced with [C_10_Nic]Br inhibitor-containing solution, the responses at different overtones corresponded rapidly and showed a much-reduced magnitude (in normalized value).

Furthermore, an increase in ΔFWHM suggests a broadening of the resonance peak, which indicates a more dissipative (viscoelastic) layer, which is often accompanied by an increase in Δ*D*. Also, a decrease in ΔFWHM signifies a more rigid layer, which usually correlates with a decrease in Δ*D*. Changes in ΔFWHM should be interpreted alongside Δ*f* and ΔD. For instance: decreased Δ*f*, increased Δ*D*, and broadened FWHM suggest mass adsorption with increased viscoelasticity. Increased Δ*f*, decreased Δ*D*, and narrowed FWHM suggest mass desorption with increased rigidity.

The combined analysis of Δ*f*, Δ*D*, and ΔFWHM provides a comprehensive understanding of the mass, viscoelasticity, and correlated structural properties of the considered layers [36,39].

**Fig. 12:**
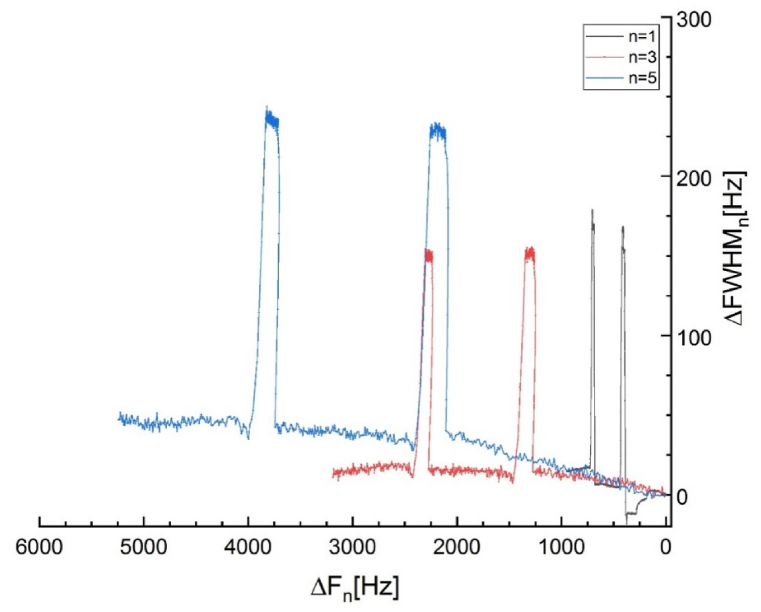
The Corresponding normalized values of ΔFWHM_n_/n vs. Δf_n_/n plot for different overtones (n = 1,3,5).

The influence of rigidity loss, which can also be detected by the frequency change (Δ*f*_n_/n), is smaller at higher overtones. This indicates that although the Sauerbrey thickness no longer relates to the full scope of the hydrated film, using the fundamental frequency (n = 1) will provide the closest value of the mass and film thickness. Significant changes in the viscoelasticity of a film or solution can also be viewed by plotting ΔFWHM against frequency change, as shown in Fig. 12.

##### 3.2.4.4 Change of dissipation vs. change of frequency (ΔD_n_ vs. Δf_n_/n)

In QCM-I investigations, the Δ*D*/Δ*f* ratio (induced energy dissipation per coupled unit mass) is commonly used to characterize the elasticity of a deposited or formed layer. Typically, a higher ΔD/Δ*f* ratio defines the development of a more elastic, dissipative coating.

Furthermore, the *f* and *D* data can be assessed by plotting Δ*f*_n_/n versus Δ*D*_*n*_. The Δ*f*-Δ*D* diagram contains some diverse points and each point represents the values of Δ*f* and Δ*D* at a certain time of the process. They essentially demonstrate how the structure of the inhibitor layer varies as a result of per-unit mass addition. Moreover, the spreading of the points relates to the kinetics of the inhibition process. A sparse distribution indicates rapid process kinetics. In summary, the Δ*f-*Δ*D* plot-trace indicates mechanistic processes, which means that a change in the direction of the trace signals that a different process is also present [22].

**Fig. 13:**
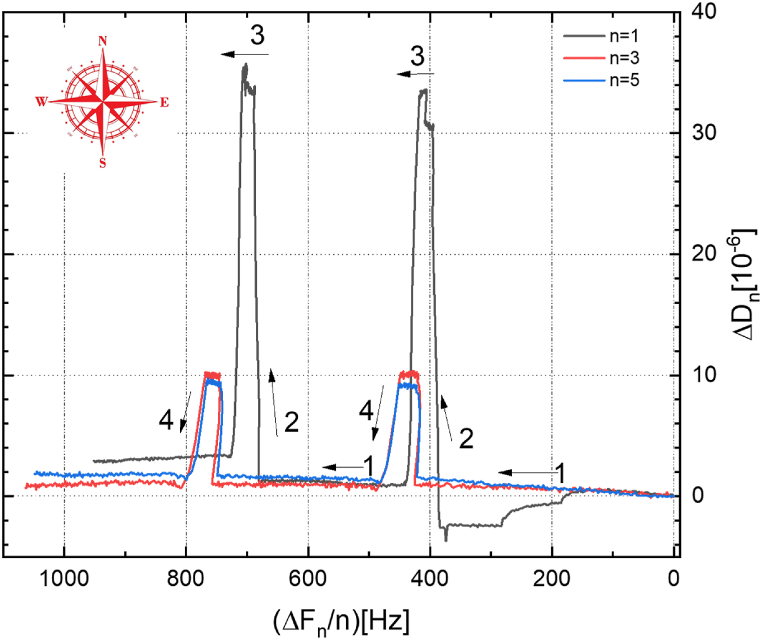
The Corresponding normalized values of Δ*D*_*n*_ vs. Δ*f*_*n*_ plot for different overtones (n = 1,3,5,9).

Fig. 13 shows the Δ*D*_n_ values of the corrosion and inhibition of Cu in 0.1 M Na_2_SO_4_ solution without and with the addition of 10^−3^ M concentration of [C_10_Nic]Br inhibitor, which is plotted as a function of Δ*f*_n_/n, for different overtones (n). However, for modeling the layers, the fundamental frequency is not commonly used as it is also the most affected by edge effects due to the finite size of the electrode. The variation in viscoelastic response becomes clearer, as displayed in Fig. 13. The step-like shaped curves instigating as a result of the superposition of shear waves in the soft layer, because of the presence of the [C_10_Nic]Br inhibitor yields that some particular value of Δ*D*_n_ might correspond to two or more different Δ*f*_*n*_ values, beside an increased mass load, may result in an increased Δ*f*_*n*_ instead of a decrease, which is projected from a rigid or semi-rigid layer (Fig. 13), [34]. To help interpret the Δ*f* and Δ*D* alterations during different phases throughout the dynamic corrosion and inhibition processes shown in Fig. 13 by arrows (labeled 1,2,3,4), an N-S-E-W lingo is used.

The whole process can be further validated in the Δ*f-*Δ*D* plots. Here, the Δ*f*_*n*_*/n* values are plotted in reverse order on the horizontal x-axis to reflect a mass increase by a negative frequency change, (as –Δ*f*_*n*_/n ≌ Δ*m*_*n*_), and Δ*D*_*n*_ values are plotted directly on the vertical y-axis presenting the change of viscoelasticity of the formed layer. Thus, the N-S-E-W model is applied to understand the Δ*f*_n_ and Δ*D*_*n*_ (i.e. mass and viscoelasticity) deviations during the dynamic corrosion and inhibition process (Fig. 13). That is, for a dynamic process directing toward the E (i.e. increase in –Δ*f*_*n*_), mass is added to the resonator surface; on the other hand, if the process is pointing W, Δm_n_ is decreasing. Alternatively, for a process pointing N (i.e. increase in Δ*D*) or S (decrease in Δ*D*), the layer on the surface becomes relatively softer or rigid, respectively [34].

In Fig. 13, the dissolution process stage in the first process (labeled 1) begins immediately after the injection of the 0.1 M Na_2_SO_4_ solution. It is presented by a flat W-E plateau in the Δ*f*_*n*_*/n*–Δ*D*_*n*_ plots, corresponding to a decrease in mass and practically nonexistent alteration in the viscoelasticity of the Cu surface.

Then, an inhibitor containing electrolyte injection prompts an S-W process (labeled 2) demonstrating a decrease in mass dissolution and an increase in viscoelasticity. It shows that the adsorption of the inhibitor hindered the Cu mass dissolution, and the layer became softer due to the complexing between the inhibitor and the corrosion products on the surface. This process suggests that the adsorption of inhibitor molecules forms a viscoelastic layer on the surface of Cu, which protects the copper electrode against dissolution. Moreover, the responses at different overtones spread out greatly, indicating an irregular distribution of the adsorbed inhibitor which is characteristic of the dissolution/deposition of layers on the Cu surface. Furthermore, the overlapping of Δ*f*_*n*_*/n* and Δ*D*_*n*_ at different overtones during this process indicates the homogeneity of the layer in the direction vertical to the surface [36,40].

Besides these two stages, a third stage (labeled 3) is also observed during the reinjection of the aggressive solution after injection of the inhibitor-containing solution. It is represented by a net W arrow, which refers to a sole mass decrease without much disturbance to the viscoelasticity of the inhibitor layer. The next stage (labeled 4) shows a sharp S arrow decrease of the dissipation energy and reduced reduction in the electrode mass. This process is probably related to having more water molecules diffused into and trapped between the inhibitor-corrosion products complex surface, balancing the Cu dissolution process, thus showing a lower decrease in the overall mass with a sharp S-type reduction of the dissipation energy (more rigid layer).

Reviakine and coworkers [41] proposed that a value of |Δ*D*_*n*_/(Δ*f*_*n*_)| < < 4 × 10^−7^ Hz^−1^ designates that the Sauerbrey equation for rigid films may be applied. Other investigators recommended that |Δ*D*_*n*_/Δ*f*_*n*_| > 1 × 10^−8^ Hz^−1^ indicates that viscoelastic models should be used [40]. These values arise from considering the ratio of Δ*D* and Δ*f* obtained as a solution to the viscoelastic wave equation (for n = 1, Δ*D*_1_/Δ*f*_1_ ∼ 2/f_0_ ∼ 4 × 10^−7^ Hz^−1^) when the film becomes thick and viscoelastic [42].

Quantitatively, in cycle 1- stage 1, after the injection of the 0.1 M Na_2_SO_4_ solution, the value of -Δ*f*_*n*_*/n for n = 3,5* reached a steady state rate of 420 ± 0.5 Hz. Meanwhile, the value of Δ*D*_*n*_ (except for the 1st overtone) reached a value of 0.9 ± 0.2 × 10^−^^6^, demonstrating the Cu dissolution process of a rigid surface. The slight increase in dissipation is due to the slightly accumulated corrosion products on the surface, because of the stagnation state of the electrolyte-Cu surface interface.

For stage labeled 2, upon the injection of the inhibitor-containing solution, the frequency responses decreased to some extent, but the dissipation responses increased promptly. This tendency shows an increase in mass and viscoelasticity of the surface layer, referring to a distinctive inhibitor adsorption process. The measured responses at the end of this inhibitor adsorption process are characterized by a large dissipation, i.e. Δ*D*_*n*_/Δ*f*_*n*_*/n* ∼9.5 ± 0.1 × 10^−^^6^ for n = 3 and 5, and 30.4 ± 0.2 × 10^− 6^ for the fundamental frequency (n = 1). It is worth mentioning that the obtained values show that both the frequency and dissipation responses exhibit apparent overtone dependencies that are characteristic of acoustically non-rigid (soft) layers.

On the other hand, a short plateau of stage labeled 3 shows stable dissipation with a decrease of the Δ*D*_*n*_/Δ*f*_*n*_*/n* of about 40 ± 0.5 Hz. Stage labeled 4, where the Δ*D*_*n*_/Δ*f*_*n*_*/n* decreases much lower than the rate in stage labeled 1 is an indication of the presence of the inhibitor on the Cu surface.

Cycle 2 of the measurement is almost a replica of cycle 1, with an insignificant deviation of the results of the fundamental frequency results which deviated slightly. The larger magnitude of the 1st overtone (∼3.4 × 10^−5^) may be due to the surficial swelling of the adsorbed inhibitor molecules in an aqueous solution [43].

### 3.3 Hypothetical inhibition mechanism

#### 3.3.1 Adsorption isotherm

An inhibitor's efficiency mostly depends on its adsorption capacity on the surface of Cu. Thus, it is required to identify the adsorption isotherm that can provide valued info on the interaction of the inhibitor and the Cu surface. The amount of surface coverage (*θ*) as a function of inhibitor concentration (*c*_*inh*_) was studied graphically by fitting it to the most common adsorption model (Langmuir adsorption isotherm).

According to this adsorption isotherm, *θ* is related to the inhibitor concentration, *c*_*inh*_, and adsorption equilibrium constant *K*_ads_ through the following expression:

(5) $$K_{ads}∙c_{inh}=\frac{\theta }{1-\theta }$$

where *K*_*ads*_ is the adsorption equilibrium constant. Eq. (5) is frequently reordered and used in the following form:

(6) $$\frac{C_{inh}}{\theta }=\frac{1}{K_{ads}}+c_{inh}$$

**Fig. 14:**
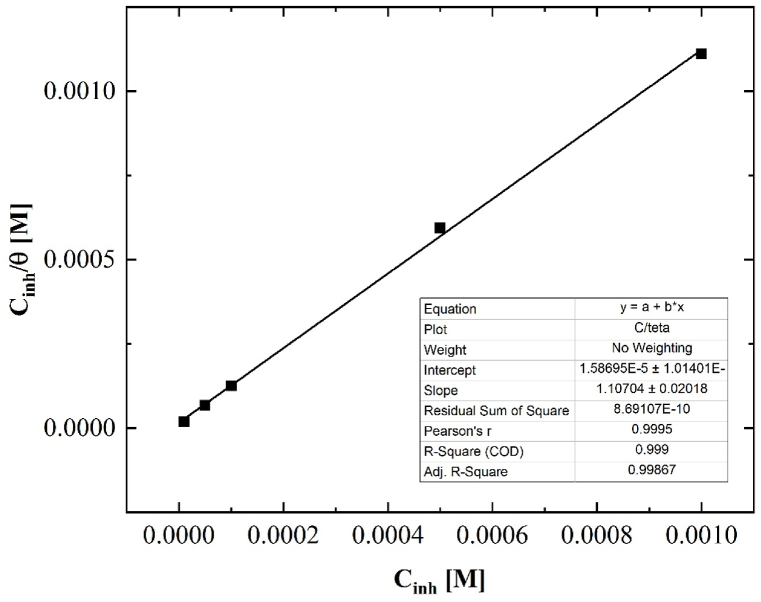
Langmuir's adsorption isotherm plot for the adsorption of the [C_10_Nic]Br inhibitor in 0.1 M Na_2_SO_4_ on Cu surface.

According to Eqs. (5), (6)), *c*_*inh*_ vs. *θ/(1-θ)* must show linear behavior and the slope of the linear plot *c*_*inh*_*/θ* vs. *c*_*inh*_ should be ≈ 1, as shown in Fig. 14.

The surface coverage was tested graphically by fitting a suitable adsorption isotherm. In the present case, the plots of *c*/*θ* versus *c* (Fig. 14) yield a straight line with the linear correlation coefficient (*R*^2^) values close to unity (0.9987), which suggests that the adsorption of the inhibitor in 0.1 M Na_2_SO_4_ medium on Cu surface follows the Langmuir adsorption isotherm more or less. The slope of the line was 1.10704, suggesting that the adsorbed molecules form a monolayer on the Cu surface and there is no interaction among the adsorbed inhibitor molecules.

The line interception was = 1.58695 × 10^−5^ which gives a value of *K*_*ads*_ = 63013 (ln *K*_*ads*_ = 11.051).

The free energy of adsorption was calculated using the following relation:

(7) $$K_{ads}=\frac{1}{55.5}e^{-\left(\Delta G_{ads}/RT\right)}$$

The calculated value of *ΔG*_*ads*_ = −37.312 kJ/mol.

Lagrenée and coworkers [44] stated that the greater the *K*_ads_ value, the more stable and stronger the adsorbed layer forming on the Cu surface which results in higher inhibition efficiency. The negative values of *ΔG*_*ads*_ show the spontaneous adsorption of the inhibitor on the Cu surface. The values of *ΔG*_*ads*_ are related to water adsorption/desorption equilibrium which forms a significant part of the overall free energy changes. In the present study, as a result of equation (7), the *ΔG*_*ads*_ value for the inhibitor is −37.312 kJ/mol, which indicates charge transfer or electron sharing from the inhibitor molecules to the Cu surface to form a coordinate type of bond [45]. The calculated standard free energy of adsorption values (−37.312 kJ/mol) was nearer to −40 kJ/mol demonstrating that the adsorption is more chemisorption than physical adsorption [45].

#### 3.3.2 Mechanism of inhibition

**Fig. 15:**
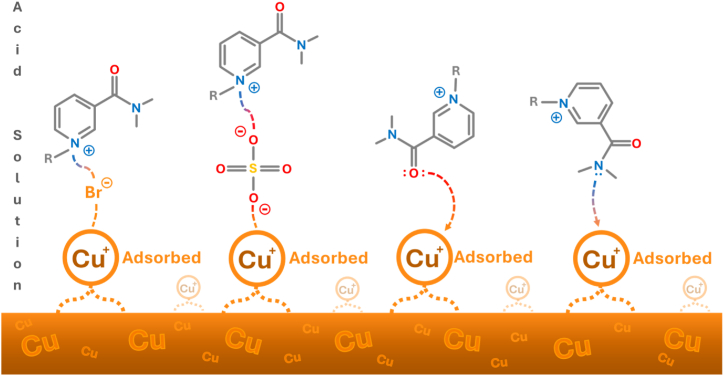
Schematic illustration of the mechanism of corrosion protection of copper by [C10Nic]Br inhibitor.

Tot and co-workers [21] used DFT calculations to examine the effect of alkyl chain length on the structure of nicotinamide. They found that in nicotinamides with an alkyl side chain longer than octyl, a more pronounced non-covalent interaction occurs between the alkyl side chain, pyridine ring, and keto oxygen, resulting in the disposition of bromide ion. Because of that, the bromide ion is located on the top of the pyridine ring, which additionally contributes to the establishment of separated hydrophobic and hydrophilic regions in the nicotinamide structures. This position of the bromide ion may play a key role in the ability of the tested ionic liquid to decrease copper corrosion in an acidic media. Namely, as is previously known, in an acidic environment on a positive copper surface the sulfate and bromide ions can be absorbed and form an interconnecting bridge between the copper ions and ILs’ cations [11,12,16,46,47]. The adsorbed anions interact with the ionic liquid cations, and thus allow cations from the ionic liquid to form a protective layer on the copper surface (Fig. 15). The strength and the type of these interactions greatly affect the inhibitory efficiency of [C_10_Nic]Br and largely depend on the length of the alkyl chain in nicotinamide, which is directly responsible for the structure of ILs.

## 4 Conclusions

• The inhibitive and adsorption activities of N-decyl nicotinamide bromide [C_10_Nic]Br against Cu corrosion in an acidic 0.1 M Na_2_SO_4_ solution (pH = 2.7) were investigated.
• The obtained results show that [C_10_Nic]Br functioned as a mixed-type inhibitor against Cu corrosion under the given conditions, whereby corrosion protection is achieved by its adsorption on the Cu surface, via anions (sulfate and bromide) that can form a linking bridge between Cu ions and the ionic liquids cations.
• The inhibition efficiency of [C_10_Nic]Br is concentration dependent, reaching η ~ 90 % at 10^−3^ M concentration.
• The time dependence of the impedance spectra supports that the adsorption of the inhibitor initially prevents copper corrosion, but later, after 5 h, the characteristics of the surface oxide layer also appear, which shows the inhibitor's effect on delaying the onset of corrosion.
• QCM-I results show that the Cu dissolution initiates instantaneously after the injection of the 0.1 M Na_2_SO_4_ solution, which corresponds to a decrease in mass and practically non-existent change in the viscoelasticity of the Cu surface. In the presence of [C_10_Nic]Br, the adsorption of the inhibitor hinders Cu dissolution, and the layer becomes softer due to the complexing between the inhibitor and the corrosion products on the surface.

## CRediT authorship contribution statement

**Gyöngyi Vastag:** Writing – review & editing, Writing – original draft, Methodology, Investigation, Formal analysis, Data curation, Conceptualization. **Ilona Felhősi:** Writing – review & editing, Writing – original draft, Methodology, Investigation, Formal analysis, Data curation, Conceptualization. **Milan Vraneš:** Resources, Data curation, Conceptualization. **Abdul Shaban:** Writing – review & editing, Writing – original draft, Methodology, Investigation, Formal analysis, Data curation, Conceptualization.

## Declaration of competing interest

The authors declare that they have no known competing financial interests or personal relationships that could have appeared to influence the work reported in this paper.
