# Supplementary material for: Impact of N-decyl-nicotineamide bromide on copper corrosion inhibition in acidic sulfate containing environment: Electrochemical and piezoelectrochemical insights
Source: Heliyon. 2024 Nov 7;10(22):e40184. doi: 10.1016/j.heliyon.2024.e40184 (PMC11583692; doi:10.1016/j.heliyon.2024.e40184)
Supplement: Multimedia component 1 [file mmc1.docx]

**Supplementary Information**

**Impact of N-Decyl-Nicotineamide Bromide on Copper Corrosion Inhibition in Acidic Sulphate containing Environment: electrochemical and Piezoelectrochemical Insights**

Gyöngyi Vastag^1^, Ilona Felhősi^2,^*, Milan Vraneš^1^, Abdul Shaban^2^

^1^University of Novi Sad, Faculty of Sciences, Department of Chemistry, Biochemistry and Environmental Protection, Trg Dositeja Obradovića 3, 21000 Novi Sad, Serbia

^2^Research Centre for Natural Sciences, Magyar tudósok körútja 2, 1117 Budapest, Hungary

Table S1: Time-variation of the fitted EIS data for copper electrode in acidic 0.1M Na_2_SO_4_ solution (pH=2.7) in the presence of 10^-3^ M [C10Nic]Br inhibitor

| **time** | **E_corr_** | **R_s_** | **Y(CPE)_f_** | **n_f_** | **R_f_** | **Y(CPE)_dl_** | **n_dl_** | **R_ct_** |
| --- | --- | --- | --- | --- | --- | --- | --- | --- |
| h | V | Ω cm^2^ | Ω^-1^ cm^-2^ s^n^ |  | Ω cm^2^ | Ω^-1^ cm^-2^ s^n^ |  | Ω cm^2^ |
| 0.5 | -0.0892 | 16.7 | - | - | - | 8.69⋅10^-5^ | 0.784 | 5963 |
| 1.4 | -0.0945 | 16.6 | - | - | - | 7.28⋅10^-5^ | 0.773 | 7458 |
| 2.4 | -0.0946 | 16.4 | - | - | - | 7.07⋅10^-5^ | 0.759 | 7814 |
| 3.3 | -0.0929 | 16.2 | - | - | - | 7.09⋅10^-5^ | 0.746 | 7933 |
| 4.3 | -0.0911 | 17.3 | 3.82⋅10^-5^ | 0.809 | 400 | 5.39⋅10^-5^ | 0.554 | 8115 |
| 5.2 | -0.0894 | 17.4 | 3.25⋅10^-5^ | 0.818 | 404 | 6.11⋅10^-5^ | 0.580 | 8233 |
| 6.2 | -0.0874 | 17.5 | 2.95⋅10^-5^ | 0.821 | 426 | 6.77⋅10^-5^ | 0.594 | 8051 |
| 7.1 | -0.0852 | 17.5 | 2.62⋅10^-5^ | 0.826 | 420 | 7.57⋅10^-5^ | 0.605 | 7827 |
| 8.1 | -0.0831 | 17.6 | 2.33⋅10^-5^ | 0.831 | 421 | 8.38⋅10^-5^ | 0.615 | 7512 |
| 9.0 | -0.0818 | 17.6 | 2.06⋅10^-5^ | 0.837 | 420 | 9.18⋅10^-5^ | 0.620 | 7243 |
| 9.9 | -0.0809 | 17.7 | 1.84⋅10^-5^ | 0.843 | 432 | 9.93⋅10^-5^ | 0.624 | 7025 |
| 10.8 | -0.0799 | 17.7 | 1.68⋅10^-5^ | 0.847 | 455 | 1.06⋅10^-4^ | 0.626 | 6826 |
| 11.8 | -0.0786 | 17.7 | 1.55⋅10^-5^ | 0.852 | 468 | 1.15⋅10^-4^ | 0.628 | 6598 |
| 12.7 | -0.0772 | 17.6 | 1.55⋅10^-5^ | 0.848 | 498 | 1.24⋅10^-4^ | 0.633 | 6309 |
| 13.7 | -0.0762 | 17.6 | 1.52⋅10^-5^ | 0.848 | 503 | 1.33⋅10^-4^ | 0.635 | 6066 |
| 14.6 | -0.0749 | 17.5 | 1.59⋅10^-5^ | 0.842 | 505 | 1.45⋅10^-4^ | 0.638 | 5760 |
| 15.6 | -0.0736 | 17.3 | 1.69⋅10^-5^ | 0.836 | 490 | 1.58⋅10^-4^ | 0.643 | 5429 |
| 16.5 | -0.0726 | 17.2 | 1.80⋅10^-5^ | 0.830 | 467 | 1.71⋅10^-4^ | 0.647 | 5127 |
| 17.5 | -0.0718 | 17.1 | 1.91⋅10^-5^ | 0.826 | 440 | 1.85⋅10^-4^ | 0.650 | 4869 |
| 18.4 | -0.0712 | 16.9 | 2.02⋅10^-5^ | 0.821 | 412 | 2.02⋅10^-4^ | 0.653 | 4705 |
| 19.4 | -0.071 | 16.8 | 2.14⋅10^-5^ | 0.817 | 392 | 2.19⋅10^-4^ | 0.657 | 4642 |
| 20.3 | -0.0711 | 16.7 | 2.20⋅10^-5^ | 0.815 | 375 | 2.36⋅10^-4^ | 0.659 | 4611 |

| **a)** | **b)** |
| --- | --- |
|  |  |
| **c)** | **d)** |
|  |  |

Figure S1. Time-variation of the corrosion potential and fitted EIS data for copper electrode in acidic 0.1M Na_2_SO_4_ solution (pH=2.7) in the presence of 10^-3^ M [C10Nic]Br inhibitor: a) corrosion potential, b) R_f_ and R_ct_ values, c) CPE elements and d) exponent of CPE elements
